# Health policy competencies in regional organizations: a retrospective analysis for 76 regional organizations from 1945 to 2015

**DOI:** 10.1186/s12992-024-01023-1

**Published:** 2024-02-26

**Authors:** Zongbin Wang, Zhisheng Liang, Xuejie Dong, Liqun Gao, Shuduo Zhou, Hui Yin, Yinzi Jin, Zhi-Jie Zheng

**Affiliations:** 1 Department of Global Health, School of Public Health, Peking University, 38 Xue Yuan Road, Haidian District, Beijing, 100191 China; 2 Institute for Global Health and Development, Peking University, Beijing, China

**Keywords:** Regional organization, Policy competencies, Health cooperation

## Abstract

**Background:**

Health policy competencies of regional organizations include mandates to create regional health laws and policies, as well as authorities that allow member states to undertake collective actions in the health field. The examination of the health policy competencies of regional organizations is essential, as it constitutes an important prerequisite for regional organizations to govern regional health. This study aims to map the development trajectory of health policy competencies in regional organizations worldwide and investigate their potential correlates. This will contribute to the enhanced promotion of both existing and new regional health cooperation.

**Methods:**

This retrospective analysis utilized the health policy competencies of the 76 regional organizations worldwide from 1945 to 2015, as investigated in the Regional Organizations Competencies Database. By aggregating member state data from various sources such as the IHME Global Burden of Disease 2019, the World Bank, and the World Trade Organization, we extracted the mean values and coefficients of variation for the covariates in regional organization characteristics, socioeconomic and demographic factors, health status and health-system capacity. The correlation between changes in the health policy scope of regional organizations and independent variables was analyzed using Poisson pseudo-likelihood regression with multiple levels of fixed effects.

**Results:**

From 1945 to 2015, the number of regional organizations with health policy competencies experienced a slow growth stage before 1991 and an explosive growth stage post-1991. By 2015, 48 out of the 71 existing regional organizations had developed their health policy competencies, yet 26 (54.2%) of these organizations possessed only 1–2 health policy competencies. An enhancement in the health policy scope of a regional organization correlated with its founding year, a greater number of policy fields, higher under-five mortality, and larger disparities in trade and healthcare access and quality indexes among member states. In contrast, larger disparities in population, under-five mortality and health worker density among member states, along with more hospital beds per capita, were negatively correlated with the expansion of a regional organization’s health policy scope.

**Conclusion:**

Since 1991, there has been a surge of interest in health among regional organizations, although health remains a secondary priority for them. The health policy competencies of regional organizations are pivotal for promoting social equity within regional communities. Its establishment is also closely linked to the level and disparities among member states in aspects such as trade, population, child mortality rates, and health system capacity.

**Supplementary Information:**

The online version contains supplementary material available at 10.1186/s12992-024-01023-1.

## Introduction

Globalization has long been studied as a driving force in the emergence of cross-border health issues and the rise of global health [1–3]. However, regional integration, closely intertwined with globalization and emerging as a response, is increasingly seen as a global trend amidst deglobalization and slow globalization [4, 5]. As a result, regional organizations have gained popularity as institutionalized forms of cooperation between three or more states based on geographical criteria, concerning more than one specific issue, with a set of primary rules and a headquarters or secretariat [6, 7]. Almost every country is a member of at least one regional organization. The profound impact of regional organizations, especially evident in their response to the COVID-19 pandemic, has unprecedentedly heightened their influence on member states and the world [8–10].

While specific research on the impact of regional integration on regional health is lacking, member states of regional organizations have empowered some organizations to create health policies and initiate regional health governance [11–13]. These health governance actions, occurring at various levels including the treaty, political, technical and intersectoral modes, set them apart from global health governance [14]. This approach offers several benefits, such as greater efficiency in reaching consensus on health policies [15, 16], integration and allocation of regional health resources [17], promotion of collective actions and the formation of regional health service and trade markets [18], and enhancement of south-south cooperation [19, 20]. Furthermore, regional organizations have emerged as vital space for global health governance, acting as a conduit for both global and national health governance and collaboration [14, 18, 21].

Recent responses to the COVID-19 pandemic have revealed deficiencies in global and national health governance [22]. However, the effectiveness of certain regional organizations indicates that the region could become a significant space in global health governance. In Europe, the early inability to coordinate and unite among member states has had serious repercussions for the European Union (EU). This has led member states to pursue a unified and coherent strategy to manage crises, resulting in the establishment of the European Health Union. As a result, some health policy authorities have been transferred from the national level to the EU [23–25]. In many developing regions, numerous countries face challenges such as fragile public health systems, severe financial constraints, and limited influence in the global medical product market and supply chain. In order to reduce the impact of these challenges on their responses to the COVID-19 pandemic, these countries have implemented regional strategies to address the epidemic [9, 18]. For instance, the African Centre for Disease Control and Prevention (Africa CDC) of the African Union and the BioDiaspora Regional Virtual Centre (ABRVC) of ASEAN aid member countries in monitoring and tracking the epidemic [26, 27]. Additionally, the African Union, ASEAN, the Andean Community (ANDEAN), and the Caribbean Community (CARICOM) have improved information sharing, connectivity in the supply chain, technological and financial cooperation, and engagement with the international community, leading to successful outcomes [8–10, 28]. Moreover, in these regions where external aid is crucial, these regional organizations have mobilized global support through establishing funds and other methods, established mechanisms and platforms for joint procurement and fair distribution [8, 27, 28]. These measures not only effectively assisted member states in combating the epidemic, but also provided new opportunities to strengthen their authority and regionalism [28]. The unique role of regional organizations, particularly in their high political level, bridging roles and coordinating across multiple sectors, is more dynamic than other levels of global health governance. Therefore, it is important to consider the potential benefits of regional organizations in global health governance.

Some studies have analyzed the driving factors, environment, and establishment processes of regional organizations that develop health policies [29–33]. Others have explored the operating framework, types of cooperation, and impact pathways of regional organizations' health cooperation regimes in these organizations [14, 17, 18]. Case analyses, such as those focusing on the South American Union's role in transmitting transnational health policies, and African regional organizations' support for regional health research cooperation, have been conducted [34, 35]. Further research has compared multiple regional organizations in sub-regions or across regions, highlighting their similarities and differences in regional health cooperation [19, 29, 36, 37]. However, these studies, primarily qualitative case studies of one or several regional organizations, have left the global panorama of regional health cooperation unclear. Specifically, it remains uncertain how many regional organizations have the authority to develop and implement health policies, and what factors influence their authority in the health field.

This issue concerns the policy competencies of regional organizations. In the field of international law and international organizations, competency is commonly used to refer to the legal authority of an international organization to deal with a particular matter [38–41]. This is because the competencies of international organizations are granted by the member states that create them. Therefore, unlike the general action competence of a country, an international organization can only act when granted authority by its member states [42]. The approach of granting authority is primarily outlined in the legal documents of the organization, such as the charter, treaties, and agreements. Specifically, The health policy competencies of regional organizations were defined as mandates to create regional health laws and policies, as well as authorities that allow member states to undertake collective actions in the health field [6, 7]. The examination of the health policy competencies of regional organizations is essential, as it constitutes an important prerequisite for regional organizations to develop health policy instruments, which is a core element of global health governance [6].

This research aimed to retrospectively analyze the development trajectory of health policy competencies and their potential correlates across 76 regional organizations from 1945 to 2015. We investigated several factors based on previous shreds of evidence and explored their associations with the health policy competencies of regional organizations. The variables explored were related to the characteristics of regional organizations, the average level and variation trends of social and economic factors, health-system capacity indicators, and disease mortality indicators among member states.

## Methods

### Data source

Based on our operational definition of regional organizations, the research objects were chosen according to three criteria: 1) consisting of at least three countries, determined by specific geographical criteria; 2) having a headquarters or secretariat; and 3) concerning more than one specific issue. Consequently, regional entities in a single field like the regional committee of the World Health Organization were omitted. Ultimately, within the available dataset, a total of 76 regional organizations were identified.

This research focused on whether regional organizations possess health policy competencies and the scope of these competencies in the health policy field. These outcomes, alongside institutional rules and characteristics of regional organizations, were sourced from the Regional Organizations Competencies (ROCO) Database, developed by Diana Panke and Anna Starkmann at Freiburg University, Germany [43]. Additional, social, economic, demographic, disease mortality and health-system capacity indicators relevant to health policy competencies were extracted from country-level values provided by IHME’s Global Burden of Disease (GBD) 2019 program, the World Bank, the World Trade Organization, among others (Table 1). They were calculated in two ways: the population-weighted average and the weighted coefficient of variation for member states. The panel database was constructed using R 4.1.3.

**Table 1:** Variable and covariates used in the analysis

Covariates	Units	Temporal coverage	Spatial coverage	Data source	Notes
Regional organization’s characteristics					
Macro region	NA	1945–2015	Regional organization	ROCO	Including Europe, America, Asia and Africa
Regional court	Yes = 1	1945–205	Regional organizations	ROCO	Representing the level of authorization of regional organizations
Age of regional organization	Years	1945–2015	Regional organization	ROCO	NA
Founding year	Yes = 1	1945–2015	Regional organization	ROCO	NA
Number of member states	NA	1945–2015	Regional organization	ROCO	NA
Decision-making rule	Yes = 1	1945–2015	Regional organization	ROCO	Representing the autonomy level of regional organizations
Policy scope	NA	1945–2015	Regional organization	ROCO	NA
Policy fields	1 to 11	1945–2015	Regional organization	ROCO	NA
Socioeconomic and demographic factors					
Scoio-demographic Index	Index	1950–2019	National to regional	GBD 2019	Mean and CV
GDP per capita	2020 USD	1950–2019	National to regional	GBD 2019	Population-weighted mean and CV
KOF globalization index	index	1970–2022	National to regional	KOF Swiss Economic Institute	Mean and CV
Trade	Million USD	1948–2021	National to regional	WTO	Sum and CV
Population	NA	1950–2019	National to regional	GBD 2019	Mean and CV
Age stracture	Percentage of population age 15–64 (%)	1960–2020	National to regional	World Bank	Population-weighted mean and CV
Continuous	Number	1950–2018	National to regional	GBD 2019	Sum and CV
Health status					
Life expectancy at birth	Years	1950–2020	National to regional	GBD 2019	Population-weighted mean and CV
Under-five mortality rate	deaths per 1000 live births	1950–2019	National to regional	GBD 2019	Population-weighted mean and CV
Age-standardized mortality of all causes	Deaths per 100,000 people	1990–2019	National to regional	GBD 2019	Population-weighted mean and CV
Health-system capacity					
Healthcare access and quality index	Index	1980–2019	National to regional	GBD 2019	Mean and CV
Hospital beds per capita	Hospital beds per 1000 people	1980–2019	National to regional	GBD 2019	Population-weighted mean and CV
Health worker density	Number of employed health workers (of any specialty) per 10,000 population	1980–2019	National to regional	GBD 2019	Population-weighted mean and CV

*Abbreviations: ROCO* regional organizations competencies database, *GBD* Global Burden of Disease *CV* coefficient of variation, *GDP* Gross Domestic Product, *WTO* world trade organization

### Health policy scope

International organization studies usually research policy competencies from two different dimensions, the scope of policy competencies (from the horizontal dimension) and the size of policy competencies (from the vertical dimension) which are composed of autonomy and bindingness [6, 44–46]. Research on the policy competencies of international organizations in the past has primarily consisted of theoretical and case studies, such as the study on competencies of the European Union [47]. As international organizations have proliferated and operated over a long time, they have amassed a substantial body of textual materials, including laws, policies, position papers, speeches, and communications. These materials serve as vital sources of information for investigating the competencies of international organizations. By leveraging information technology and text analysis, researchers can directly analyze these materials, organize them into structured data and information, and even utilize them to develop indices. This process offers quantitative possibilities for examining the authority of international organizations [48]. This study focuses on the scope of health policy competencies for regional organizations, as the legal texts where policy competencies are from are relatively standardized, which helps to produce structured data.

The ROCO Database systematically compiles structured information on 76 regional organizations from 1945 to 2015 in an organization-year-format, including four sub-databases (I, II, III and IV). The ROCO I database charts health policy competencies of regional organizations based on their main treaties during this period, covering 14 specific policy competencies (or sub-categories): food safety, disease, disease prevention, drugs, epidemic/pandemic, health, health care/health services/health system, medical, mortality, outbreak, public health, sanitary, vaccine /vaccination, and wellbeing. These competencies are identified by a theory-driven clustering of coding buzzwords, utilizing a multi-stage inductive strategy in the coding scheme. The ROCO I database also differentiates between external and internal domains for each policy competence, based on buzzwords that define the context of the application. Hence, the health policy scope of a regional organization, conceptualized as the number of different health policy competencies it covers in a given year, can theoretically range from 0 to 28, including 14 internal and 14 external health policy competencies [6].

### Hypotheses and variable selection

To examine the factors influencing the health policy competencies of regional organizations, four hypotheses were established drawing insights from prior literature. The first hypothesis relates to the characteristics of regional organizations, encompassing aspects like their age, the number of member states, decision-making rules, and authorization. These factors have been associated with changes in the policy scope of regional organizations [46, 49]. The second hypothesis focuses on the socioeconomic and demographic factors of member states, as they are the foundational aspects of regional integration and also the socioeconomic, commercial and political determinants of health, which can drive the interface of regional integration and health policy [14, 45, 50]. These include the social demographic index (SDI), GDP per capita, globalization index, import and export trade, population, age structure and continuity. The third and fourth hypotheses are directly related to health, which must be taken into account when member states develop policy competencies of a regional organization. The third hypothesis addresses the health status of member states, encompassing life expectancy at birth, under-five mortality rate, and age-standardized mortality of all causes. The fourth hypothesis examines the health-system capacity of member states, represented by the Healthcare Access and Quality Index (HAQI), hospital beds per capita, and health worker density. Due to limited data availability, indicators related to health expenditures were not included in our study (Table 1). To account for the time needed to influence policy competencies, we applied a two-year lag to all covariates in hypotheses two to four.

### Statistical analysis

In this study, a total of 2291 records were generated from 76 regional organizations spanning the period from 1945 to 2015. The analysis was structured in three stages: the first stage focused on revealing the temporal and spatial distributions of health policy competencies among regional organizations. The second stage involved analyzing the correlation between the health policy scope of regional organizations and their policy fields and policy scope in 2015. In the third stage, to explore the four previously mentioned hypotheses, the first-order difference in health policy scope was used as the dependent variable. Given the nature of the dependent variable as discrete and heteroscedastic, Poisson pseudo-likelihood regression with multiple levels of fixed effects and clustering standard error was employed for the analysis [51].

For comparative purposes, all continuous variables were centred at 0 and scaled to have a standard deviation (SD) of 1. Due to the covariates being at different levels of the causal chain, highly correlated, and some containing numerous missing values, regression analyses for each hypothesis were conducted individually with time and two-way fixed effects. To address the issue of multiple hypothesis testing, the Bonferroni correction was applied. This adjusted the covariates and expected significance level to 0.05, resulting in a significance cutoff value of 0.0125, considering each indicator represented one of the four hypotheses (*N* = 4). All analyses were performed using Stata 17.0.

## Results

**Fig. 1:**
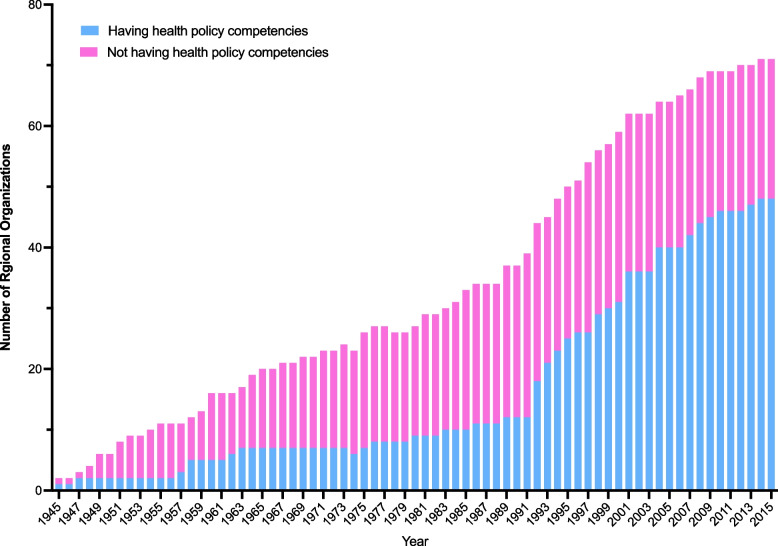
Number of regional organizations and those with health policy competencies from 1945 to 2015

Between 1945 and 2015, 51 (67.1%) of the 76 regional organizations had developed policy competencies in health (eTable 1). The evolution of these competencies can be categorized into two stages: 1) Slow Growth Stage (1945–1991): During this period, the number of regional organizations gradually increased. By 1991, only 12 out of 39 (30.8%) organizations had developed health policy competencies. 2) Explosive Growth Stage (Post-1991): Since 1991, there has been a significant increase in both the number of regional organizations and those with accompanying health policy competencies (Fig. 1).

**Fig. 2:**
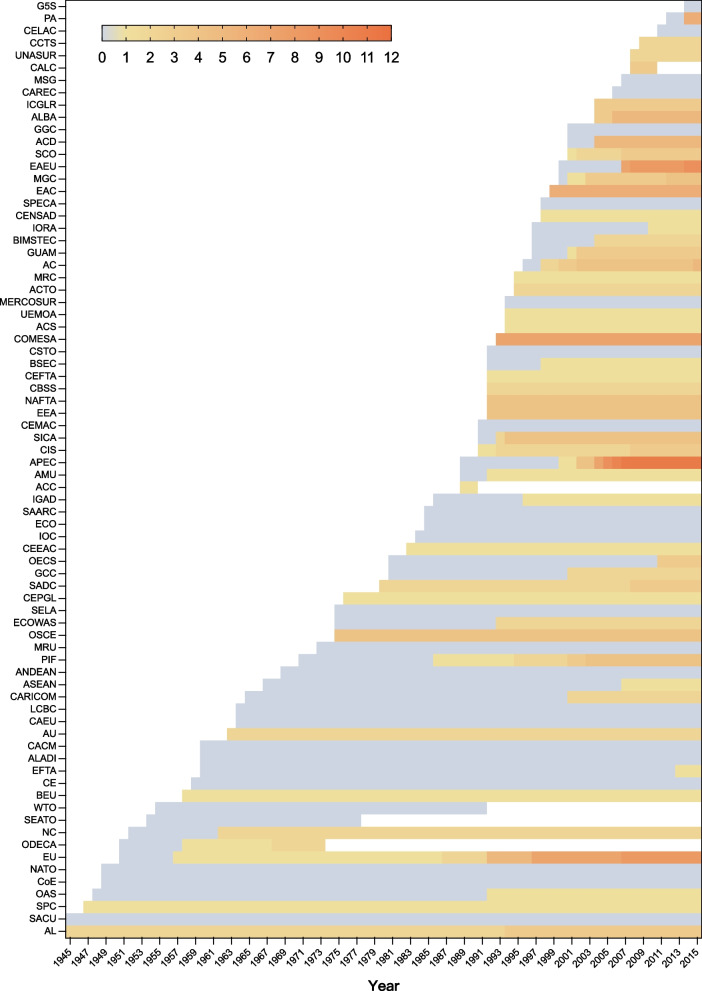
The development trajectory of health policy competency scope in 76 regional organizations from 1945 to 2015

From 1945 to 2015, 52.9% (27 out of 51) of regional organizations included health policy competencies in their primary laws at their inception. The remaining 47.1% (24 regional organizations) adopted these competencies over time, with the European Free Trade Association (EFTA) incorporating them after 53 years. Of these organizations, 37 (72.5%) have not altered their health policy competencies since their introduction. However, 14 organizations, including the Asia–Pacific Economic Cooperation (APEC), the European Union (EU), and the Eurasian Economic Union (EAEU), expanded their scope of health policy competencies. Notably, the EU's competencies grew from one in 1957 to eight in 2015, and APEC's from one in 2000 to 11 in 2015 (Fig. 2).

**Fig. 3:**
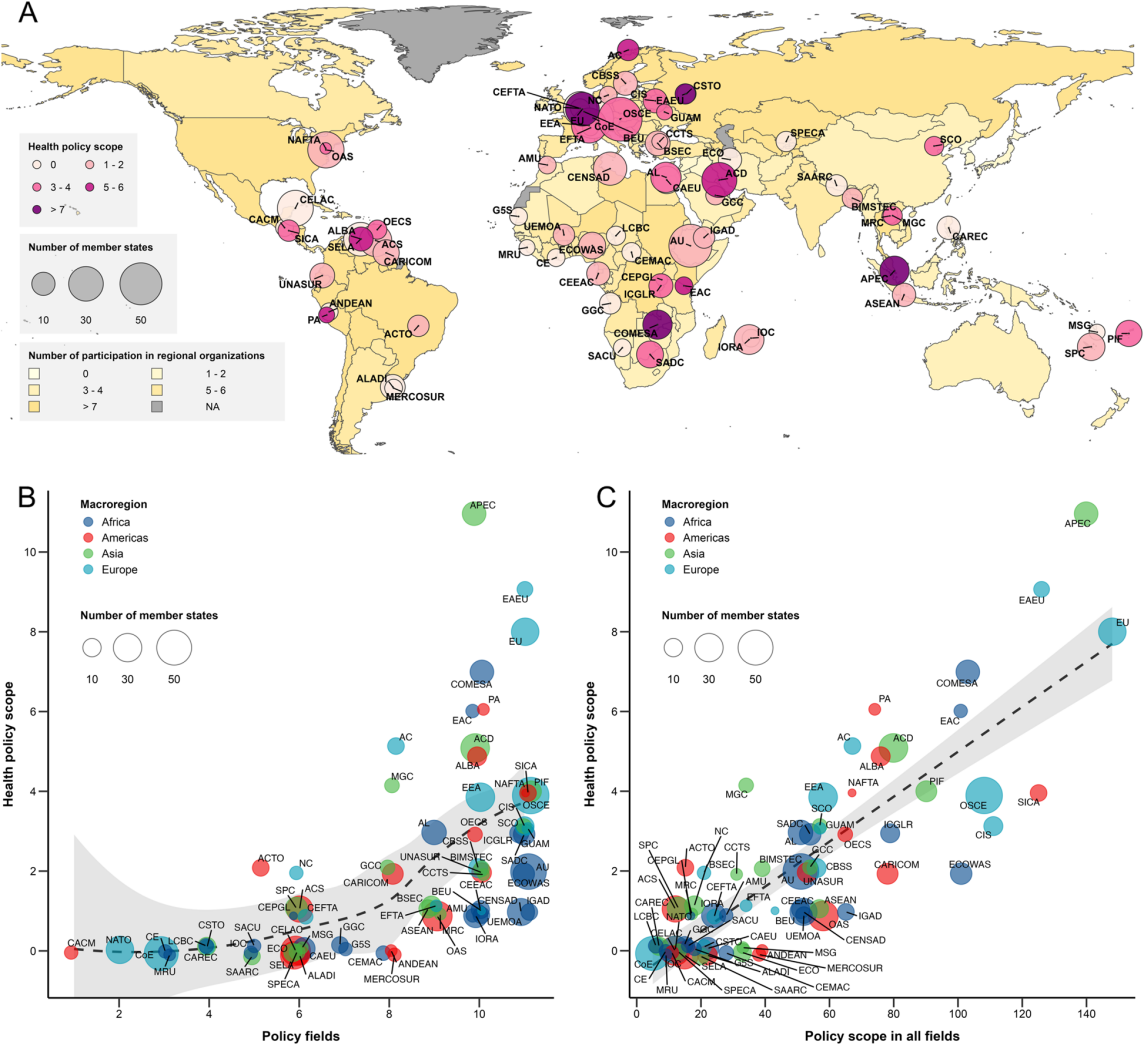
Health policy competency scope and its relationship to policy fields and policy scope of all fields in 71 regional organizations in 2015. The A shows the health policy scope of the 71 regional organizations in 2015. The center of the circle is the location of the regional organization secretariat, the size of each circle indicates the number of member States, and the colour of the map indicates the number of regional organizations each country participates in. B shows the relation between regional organizations' health policy scope and policy fields. C shows the relationship between their health policy scope and overall policy scope. The different colours indicate the macro area where the regional organization secretariat is located

In 2015, of the 71 existing regional organizations, 67.6% (48) had developed health policy competencies. These organizations were distributed globally without significant regional differences. However, the scope of health policy competencies varied significantly: 54.2% (26 out of 48) had only 1–2 health policy competencies, while APEC, EAEU, and EU had the most, with 11, 9, and 8 competencies, respectively (Fig. 3A). Additionally, 93.8% (45 out of 48) of these organizations possessed only internal health policy competencies. Only three organizations, namely APEC, Pacific Islands Forum (PIF), and Bolivarian Alliance for the Peoples of Our Americas (ALBA), had both internal and external health policy competencies (eTable 2).

Correlations were observed between the health policy competency scope of regional organizations and their policy fields, as well as their policy scope in 2015. Regional organizations with fewer policy fields tended to have underdeveloped health policy competencies. In contrast, those with five or more policy fields were more inclined to develop these competencies, and as their policy fields expanded, so did their health policy scope. This trend is illustrated in Fig. 3B. Additionally, organizations with a limited policy scope across all fields often lacked health policy competencies. However, when a regional organization's policy scope reached or exceeded 40, health policy competencies were universally included, showing a linear increase in scope, as depicted in Fig. 3C.

**Fig. 4:**
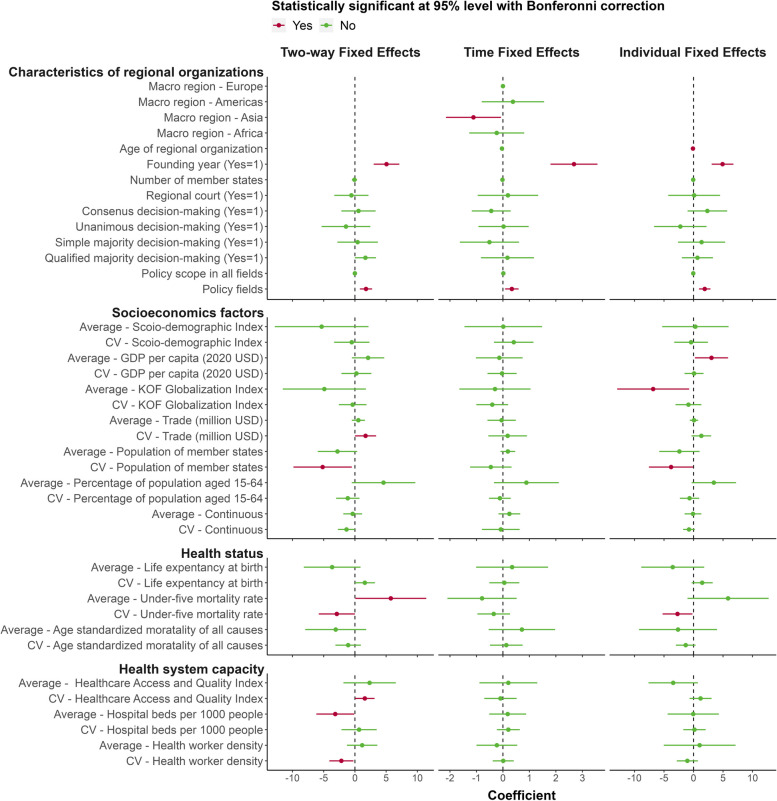
The association between organizational characteristics, socioeconomic and demographic factors, health status and health system capacity and the growth in the health policy competency scope of regional organizations. Poisson pseudo-likelihood regression with multiple levels of fixed effects was used for analysis. The left column shows the estimated associations of key factors with changes in the health policy scope of regional organizations by the two-way fixed effects regression model. The middle column shows the estimated associations of the time-fixed effect regression model. The right column represents the estimated associations of the individual fixed effect regression model. Red indicates the association is not significant, and green indicates the association is significant at a 95% CI with a Bonferroni correction. CV, coefficient of variation

The two-way fixed effects model regression analysis addresses changes in the health policy scope of regional organizations, taking into account their organizational characteristics, socioeconomic and demographic factors, population health status and the health-system capacity of member states. This analysis unveiled several critical insights: Regional organizations were more likely to establish their health policy competencies in their founding year. An expansion in policy fields was associated with an increase in health policy scope change. Greater disparities in trade and smaller population gaps among member states correlated with a more significant change in health policy scope within the region. Regions with higher under-five mortality rates were linked to a broader health policy scope of regional organizations. Conversely, larger disparities in these rates were tied to less health policy scope change. A greater variation in the Healthcare Access and Quality Index (HAQI) among member states positively influenced the increase in health policy scope. More hospital beds per thousand population in a region and a larger variation in health worker density among member states corresponded to smaller changes in health policy scope. These findings are detailed in Fig. 4 and eTable 3.

## Discussion

This research represents the first global analysis of health policy competencies for 76 regional organizations, based on their primary legal documents spanning from 1945 to 2015. The findings reveal that before the 1990s, health policy was not a major focus for regional organizations, but a marked increase in interest emerged in the subsequent period. This shift can be attributed to the evolution of regionalism’s driving forces. The first wave of regionalism (1945–1990) was propelled by the desire to avoid war and pursue economic interests amid the Cold War. In contrast, the second wave (1990–2015) responded to the expanding global disparities promoted by globalization and free trade policies, particularly in social inequality, including health, and regional social risks [6, 52, 53]. In response to these changing dynamics, regional organizations began to shift their focus from traditional economic objectives to a more holistic approach that emphasizes social welfare and soft power. [15, 31, 54] Consequently, health emerged as a crucial policy field for regional organizations, reflecting a vital aspect in the social sector.

However, the extent to which different regional organizations prioritize health varies, linking it to diverse value views, such as security, development, trade, human rights, ethics and even moral standards [29, 55, 56]. Although this framing of health has contributed to incorporating health into the insight of regional power institutes, it has also inadvertently undermined the importance of health itself. This may impede the autonomous discourse on health, which consequently relegates health to a secondary priority area for most regional organizations. Our findings also indicate that the top four policy areas prioritized by these organizations typically do not include health, which often ranks between the 8th and 11th. Additionally, the proportion of health policy competencies within the overall policy competency scope of most regional organizations is also relatively low. These findings may be attributed to the perception that health is solely regarded as a subset of social policy in regional organizations. In these organizations, particularly in developing nations, the relative superiority of health in social policies is difficult to be apparent, given limited resources needing to be allocated in various social areas which include environment, migration, development, science and technology, education, health and others. This observation aligns with various case studies, particularly those focusing on regional organizations in Africa and Latin America [35, 56–58]. However, the Economic Community of West African States (ECOWAS) is a positive example. It established a regional health institution, the West African Health Organization (WAHO), to respond to severe public health crises like Ebola faced long in this region, which provided an extra promotional factor to raise the priority of health [59]. In addition to its high professionalism, WAHO possesses several distinguishing features: 1) WAHO maintains a relative level of independence, setting it apart from the health regimes of other regional organizations; 2) WAHO holds a high political status, with its highest decision-making body being the Council of Heads of State and Government. This sets it apart from regional offices of the World Health Organization, which makes decisions at the level of the Ministry of Health; and 3) WAHO exhibits cross-departmental characteristics, with its decision-making body including departments for regional integration, finance, and planning, distinguishing it from specialized technical institutions such as the European Centre for Disease Prevention and Control and the Africa Centres for Disease Control and Prevention [60].

The analysis of the first hypothesis did not find that certain characteristics of regional organizations, such as the presence of regional courts, majority decision-making, and a larger number of member states, contribute to the expansion of health policy competencies. However, other studies using the same dataset suggest this contribution was evident in the policy scope in all fields [6, 61]. This is due to the low proportion and insignificant differences in health policy in regional organizations failing to adequately reflect the variation among organizations, despite bindingness, autonomy and size of regional organizations being some important factors [46, 62]. Instead, this study shows that the establishment of health policy competencies at a regional organization’s inception appears to be a crucial factor in determining its future scope in this field. Organizations in the early stages of establishment possess more flexibility to explore new fields. Conversely, more mature regional organizations, having developed over the years, face challenges in introducing new fields like health. This difficulty arises from path dependence and a tendency to maintain the status quo bias over time [6, 61, 63].

The finding of the second hypothesis emphasizes that the trade and population disparities among member states are significant socio-economic and demographic factors influencing the regional organizations to formulate health policy competencies. The strong linkage between globalization, regional integration, and trade underscores the importance of trade in the development of regional integration. The dual impact of trade on health, particularly in regional trade scenarios, has led some regional organizations to establish health policies [14, 64, 65]. An increasing trade gap between member states can negatively affect regional integration and development, prompting regional organizations to focus on policy impact in social sectors like health. This is especially true when regional powers advocate for policies to support vulnerable member states against the potential negative impacts, including health risks [44, 66].

However, we also find that significant differences in population size between member states can hinder collaborative regional efforts and resource allocation, ultimately impeding the development of health policy competencies within regional organizations. In general, large countries exhibit a stronger inclination to engage in regional health cooperation due to the wider impact of external influences on their population. And their relatively abundant human resources also enable greater pursuit of regional health actions. Conversely, small countries have more limited ability than their larger counterparts to fulfill certain obligations with regional organizations, such as payment contributions and joint actions. These challenges can discourage their participation in regional health cooperation. Additionally, small states often unite to safeguard themselves against neighbouring regional powers, fostering a greater willingness among them to develop health policy competencies among themselves, rather than with larger states [44].

For the third hypothesis, this research also identified a high under-five mortality rate as a key factor in promoting regional health policies. Nevertheless, large disparities in this rate among member states could obstruct the growth of health policy competencies. The under-five mortality rate is not only a comprehensive indicator to represent a nation’s health, but also a crucial aspect of archiving the Millennium Development Goals and Sustainable Development Goals [67–69]. High mortality rates among children under five encourage member states to cooperate and take collective actions for reduction. Yet, for low- and middle-income countries, healthcare resources to improve child mortality are mainly from the health assistance of developed countries. Although certain member states may have lower child mortality, they currently lack the capacity to assist fellow member countries in a common regional organization. Therefore, there is a wide variance in these rates among member states, but those with better performance may be less inclined to cooperate in this area [70, 71].

Furthermore, the results of the fourth hypothesis highlight the necessity of adhering to common norms and unified standards in the healthcare systems to facilitate cross-border access to consistent and essential healthcare services among member states. Significant disparities in healthcare accessibility and quality among member states necessitate strengthened coordination and cooperation in health policy to advance regional integration [72]. It also revealed that inadequate health infrastructure and large disparities in the health workforce can hinder health cooperation among member states. This is particularly challenging for regional organizations in the global south, which may struggle to drive cooperation without additional external support, such as the Central American Integration System (SICA) and others [72–74]. In these regions that heavily rely on health development assistance, these inherent objective factors impose limitations on the enhancement of regional health policy competencies [75].

The health policy competencies are important as they provide regional organizations legitimacy to address national, regional and global health challenges. Future studies will also aim to understand the activated conditions of health policy competencies, their operational mechanisms, and their outcomes of policy-making and collective actions. It is evident that organizational characteristics or regional organizations, as well as socio-economic factors, health status and health system capacity of member states, will continue to be taken into consideration. These factors will interact with the health policy competencies of regional organizations, potentially influencing regional and global health governance. Furthermore, a significant area of research will also be the impact of regional health policies on the behaviour of member states' health governance, which aims to determine whether these policies will have a positive effect on member states' health outcomes and convergence, ultimately promoting regional health development and equity.

## Limitations

This research has several limitations: 1) The health policy competencies were identified using the ROCO Database, which includes only the primary laws of regional organizations. This approach might not fully capture the health actions implemented by some regional organizations that are not reflected in their charters. 2) The determination of policy competencies through text keyword cluster analysis may not fully align with professional classification. The applicability of this method in this research context should be considered. 3) The covariates were sourced from multiple origins, with some data estimated by models, potentially limiting their quality and interpretability. 4) the covariates have varying time ranges and may not comprehensively cover all periods of regional organizations’ existence. The average value and variation coefficient for regional organizations were calculated using national data, which may not accurately estimate the certainty of the data. This could challenge the results of the regression model. 5) The study’s exploratory analysis of the influencing factors does not provide an internal explanatory mechanism. 6) This study focuses on the period from 1945 to 2015 and does not take into account changes in the past decade. However, both this study and related literature indicate that the number of regional changes has been relatively stable from 2010 to 2020, and the expansion of policy scope has similarly stabilized [76]. Therefore, the conclusions drawn from this study may still be applicable after 2015. However, it is important to further consider the impact of the changes since the COVID-19 pandemic.

Regional health cooperation is highly politicized. However, quantifying and revealing the influence of political, diplomatic, and unexpected events poses significant challenges [77]. Therefore, it is essential to integrate qualitative interviews or case studies with quantitative research in mixed methods research. Despite the perception that health is of low political sensitivity, regional collective actions necessitate a high-level political commitment to drive progress. Factors such as the political, economic, and diplomatic relationships between member states, regional dominant powers, interference from major external powers, and major emergencies are all crucial factors that influence the ability of regional member countries to achieve political consensus. Research on these issues requires in-depth interdisciplinary studies of health, international relations, and diplomacy. Additionally, the nature, structure, and operational models of different regional organizations vary, which ultimately affects the approaches and intensities of regional collective actions, thus presenting inconsistent impacts on regional health improvement and convergence. Hence, identifying sufficiently excellent regional organization cases remains critical.

## Conclusion

Since the 1990s, the global challenges posed by globalization, particularly regarding inequality, have spurred a surge of interest in health among regional organizations. Despite this increased focus, health remains a secondary priority for these organizations, primarily serving only as a byproduct of social development. The establishment and expansion of health policy competencies in regional organizations are crucial for promoting social equity within regional communities. These competencies are intimately connected to the level and disparities among member states in aspects like trade, population, child mortality rates, and health system capacity.

### Supplementary Information

**Supplementary Material 1. **

## Data Availability

All the data in the study can be fully accessed in open databases.

## Abbreviations

IHME: Institute for Health Metrics and Evaluation
ROCO: Regional Organizations Competencies Database
GBD: Global Burden of Disease
SDI: Social Demographic Index
GDP: Gross Domestic Product
HAQI: Healthcare Access and Quality Index
AC: Arctic Council
ACC: Arab Cooperation Council
ACD: Asia Cooperation Dialogue
ACS: Association of Caribbean States
ACTO: Amazonian Cooperation Treaty Organization
AL: Arab League
ALADI: Latin American Integration Association
ALBA: Bolivarian Alliance for the Peoples of Our Americas
AMU: Arab Maghreb Union
ANDEAN: Andean Community
APEC: Asia–Pacific Economic Cooperation
ASEAN: Association of South East Asian Nations
AU: African Union
BEU: Benelux Economic Union
BIMSTEC: Bay of Bengal Initiative for Multi-Sectoral Technical and Economic Cooperation
BSEC: Black Sea Economic Cooperation
CACM: Central American Common Market
CAEU: Council of Arab Economic Unity
CALC: Latin American and Caribbean Summit on Integration and Development; CAREC: Central Asia Regional Economic Cooperation
CARICOM: Caribbean Community
CBSS: Council of the Baltic Sea States
CCTS: Cooperation Council of Turkic Speaking States
CE: Conseil de l'Entente
CEEAC: Communaute Economique des etats de l'Afrique Centrale
CEFTA: Cental European Free Trade Agreement
CELAC: Community of Latin American and Caribbean States
CEMAC: Communaute Economique et monetaire de l'Afrique centrale
CENSAD: Community of Sahel-Saharan States
CEPGL: Economic Community of the Great Lakes Countries
CIS: Commonwealth of Independent States
CoE: Council of Europe
COMESA: Common Market for Eastern and Southern Africa
CSTO: Collective Security Treaty (Organization)
EAC: East African Community
EAEU: Eurasian Economic Union
ECO: Economic Cooperation Organization
ECOWAS: Economic Community of West African States
EEA: European Economic Area
EFTA: European Free Trade Association
EU: European Union
G5S: G5 du Sahel
GCC: Gulf Cooperation Council
GGC: Gulf of Guinea Commission
GUAM: Organization for Democracy and Economic Development
ICGLR: International Conference on the Great Lakes Region
IGAD: Intergovernmental Authority on Develeopment
IOC: Indian Ocean Commission
IORA: Indian Ocean Rim Association
LCBC: Lake Chad Basin Commission
MERCOSUR: Mercado Commun del Sur
MGC: Mekong-Ganga Cooperation
MRC: Mekong River Commission
MRU: Mano River Union
MSG: Melanesian Spearhead Group
NAFTA: North American Free Trade Organization
NATO: North Atlantic Treaty Organization
NC: Nordic Council
OAS: Organization of American States
ODECA: Organization of Central American States
OECS: Organization of Eastern Caribbean States
OSCE: Organization for Security and Cooperation in Europe
PA: Pacific Alliance
PIF: Pacific Islands Forum
SAARC: South Asian Association for Regional Cooperation
SACU: Southern African Customs Union
SADC: Southern African Development Community
SCO: Shanghai Cooperation Organization
SEATO: Southeast Asia Treaty Organization
SELA: Latin American Economic System
SICA: Central American Integration System
SPC: Pacific Community
SPECA: UN Special Program for the Economies of Central Asia
UEMOA: West African Economic and Monetary Union
UNASUR: Union of South American Nations
WTO: Warsaw Treaty Organisation
