# Supplementary material for: Health policy competencies in regional organizations: a retrospective analysis for 76 regional organizations from 1945 to 2015
Source: Global Health. 2024 Feb 26;20:17. doi: 10.1186/s12992-024-01023-1 (PMC10895825; doi:10.1186/s12992-024-01023-1)
Supplement: Supplementary file 1 — Supplementary Material 1. [file 12992_2024_1023_MOESM1_ESM.docx]

**Supplemental Online Content**

**eTable 1.** The abbreviation, full name, founding year, year of health policy competencies establishment and year of dissolution of the 76 regional organizations in the ROCO database

**eTable 2.** Health policy scope, policy scope and policy fields of regional organizations with health policy competencies in 2015

**eTable 3.** The association between organizational characteristics, socioeconomic and demographic factors, health status and health system capacity and the growth in the health policy competency scope of regional organizations, Coefficient (95% CI with Bonferroni correction)

This supplemental material has been provided by the authors to give readers additional information about their work.

**eTable 1** **The abbreviation, full name, founding year, year of health policy competencies establishment and year of dissolution of the 76 regional organizations in the ROCO database ^a^**

| Regional organization | Full name | Founding year | Founding year of health policy competencies | Disso-lution year |
| --- | --- | --- | --- | --- |
| AC | Arctic Council | 1996 | 1998 |  |
| ACC | Arab Cooperation Council | 1989 | 1989 | 1990 |
| ACD | Asia Cooperation Dialogue | 2001 | 2004 |  |
| ACS | Association of Caribbean States | 1994 | 1994 |  |
| ACTO | Amazonian Cooperation Treaty Organization | 1995 | 1995 |  |
| AL | Arab League | 1945 | 1945 |  |
| ALADI | Latin American Integration Association | 1960 | NA |  |
| ALBA | Bolivarian Alliance for the Peoples of Our Americas | 2004 | 2004 |  |
| AMU | Arab Maghreb Union | 1989 | 1992 |  |
| ANDEAN | Andean Community | 1969 | NA |  |
| APEC | Asia-Pacific Economic Cooperation | 1989 | 2000 |  |
| ASEAN | Association of South East Asian Nations | 1967 | 2007 |  |
| AU | African Union | 1963 | 1963 |  |
| BEU | Benelux Economic Union | 1958 | 1958 |  |
| BIMSTEC | Bay of Bengal Initiaitve for Multi-Sectoral Technical and Economic Cooperation | 1997 | 2004 |  |
| BSEC | Black Sea Economic Cooperation | 1992 | 1998 |  |
| CACM | Central American Common Market | 1960 | NA |  |
| CAEU | Council of Arab Economic Unity | 1964 | NA |  |
| CALC | Latin American and Caribbean Summit on Integration and Development | 2008 | 2008 | 2010 |
| CAREC | Central Asia Regional Economic Cooperation | 1997 | NA |  |
| CARICOM | Caribbean Community | 1965 | 2001 |  |
| CBSS | Council of the Baltic Sea States | 1992 | 1992 |  |
| CCTS | Cooperation Council of Turkic Speaking States | 2009 | 2009 |  |
| CE | Conseil de l'Entente | 1959 | NA |  |
| CEEAC | Communaute Economique des etats de l'Afrique Centrale | 1983 | 1983 |  |
| CEFTA | Cental European Free Trade Agreement | 1992 | 1992 |  |
| CELAC | Community of Latin American and Caribbean States | 2011 | NA |  |
| CEMAC | Communaute Economique et monetaire de l'Afrique centrale | 1991 | NA |  |
| CENSAD | Community of Sahel-Saharan States | 1998 | 1998 |  |
| CEPGL | Economic Community of the Great Lakes Countries | 1976 | 1976 |  |
| CIS | Commonwealth of Independent States | 1991 | 1991 |  |
| CoE | Council of Europe | 1949 | NA |  |
| COMESA | Common Market for Eastern and Southern Africa | 1993 | 1993 |  |
| CSTO | Collective Security Treaty (Organization) | 1992 | NA |  |
| EAC | East African Community | 1999 | 1999 |  |
| EAEU | Eurasian Economic Union | 2000 | 2007 |  |
| ECO | Economic Cooperation Organization | 1985 | NA |  |
| ECOWAS | Economic Community of West African States | 1975 | 1993 |  |
| EEA | European Economic Area | 1992 | 1992 |  |
| EFTA | European Free Trade Association | 1960 | 2013 |  |
| EU | European Union | 1951 | 1957 |  |
| G5S | G5 du Sahel | 2014 | NA |  |
| GCC | Gulf Cooperation Council | 1981 | 2001 |  |
| GGC | Gulf of Guinea Commission | 2001 | NA |  |
| GUAM | Organization for Democracy and Economic Development | 1997 | 2001 |  |
| ICGLR | International Conference on the Great Lakes Region | 2004 | 2004 |  |
| IGAD | Intergovernmental Authority on Develeopment | 1986 | 1996 |  |
| IOC | Indian Ocean Commission | 1984 | NA |  |
| IORA | Indian Ocean Rim Association | 1997 | 2010 |  |
| LCBC | Lake Chad Basin Commission | 1964 | NA |  |
| MERCOSUR | Mercado Commun del Sur | 1994 | NA |  |
| MGC | Mekong-Ganga Cooperation | 2000 | 2001 |  |
| MRC | Mekong River Commission | 1995 | 1995 |  |
| MRU | Mano River Union | 1973 | NA |  |
| MSG | Melanesian Spearhead Group | 2007 | NA |  |
| NAFTA | North American Free Trade Organization | 1994 | 1992 |  |
| NATO | North Atlantic Treaty Organization | 1949 | NA |  |
| NC | Nordic Council | 1952 | 1962 |  |
| OAS | Organization of American States | 1948 | 1992 |  |
| ODECA | Organization of Central American States | 1951 | 1958 | 1973 |
| OECS | Organization of Eastern Caribbean States | 1981 | 2011 |  |
| OSCE | Organization for Security and Cooperation in Europe | 1975 | 1975 |  |
| PA | Pacific Alliance | 2012 | 2014 |  |
| PIF | Pacific Islands Forum | 1971 | 1986 |  |
| SAARC | South Asian Association for Regional Cooperation | 1985 | NA |  |
| SACU | Southern African Customs Union | 1945 | NA |  |
| SADC | Southern African Development Community | 1980 | 1980 |  |
| SCO | Shanghai Cooperation Organization | 2001 | 2001 |  |
| SEATO | Southeast Asia Treaty Organization | 1954 | NA | 1977 |
| SELA | Latin American Economic System | 1975 | NA |  |
| SICA | Central American Integration System | 1991 | 1993 |  |
| SPC | Pacific Community | 1947 | 1947 |  |
| SPECA | UN Special Program for the Economies of Central Asia | 1998 | NA |  |
| UEMOA | West African Economic and Monetary Union | 1994 | 1994 |  |
| UNASUR | Union of South American Nations | 2008 | 2008 |  |
| WTO | Warsaw Treaty Organisation | 1955 | NA | 1991 |

Abbreviations: ROCO, Regional Organizations Competencies Database.

^a^ Data comes form <https://dataverse.harvard.edu/dataset.xhtml?persistentId=doi:10.7910/DVN/UBXZHC>

**eTable 2. Health policy scope, policy scope and policy fields of regional organizations with health policy competencies in 2015**

| Regional organization | Internal health policy scope | External health policy scope | Total health policy scope | Total policy scope | Percentage of health in total policy scope (%) | Policy field |
| --- | --- | --- | --- | --- | --- | --- |
| AC | 5 | NA | 5 | 67 | 7.5 | 8 |
| ACD | 5 | NA | 5 | 80 | 6.3 | 10 |
| ACS | 1 | NA | 1 | 12 | 8.3 | 6 |
| ACTO | 2 | NA | 2 | 15 | 13.3 | 5 |
| AL | 3 | NA | 3 | 51 | 5.9 | 9 |
| ALBA | 4 | 1 | 5 | 76 | 6.6 | 10 |
| AMU | 1 | NA | 1 | 28 | 3.6 | 10 |
| APEC | 8 | 3 | 11 | 140 | 7.9 | 10 |
| ASEAN | 1 | NA | 1 | 57 | 1.8 | 9 |
| AU | 2 | NA | 2 | 51 | 3.9 | 11 |
| BEU | 1 | NA | 1 | 43 | 2.3 | 10 |
| BIMSTEC | 2 | NA | 2 | 39 | 5.1 | 10 |
| BSEC | 1 | NA | 1 | 18 | 5.6 | 9 |
| CARICOM | 2 | NA | 2 | 78 | 2.6 | 8 |
| CBSS | 2 | NA | 2 | 56 | 3.6 | 10 |
| CCTS | 1 | NA | 1 | 31 | 6.5 | 10 |
| CEEAC | 1 | NA | 1 | 51 | 2.0 | 10 |
| CEFTA | 1 | NA | 1 | 24 | 4.2 | 6 |
| CENSAD | 1 | NA | 1 | 52 | 1.9 | 11 |
| CEPGL | 1 | NA | 1 | 17 | 5.9 | 6 |
| CIS | 3 | NA | 3 | 111 | 2.7 | 11 |
| COMESA | 7 | NA | 7 | 103 | 6.8 | 10 |
| EAC | 6 | NA | 6 | 101 | 5.9 | 10 |
| EAEU | 9 | NA | 9 | 126 | 7.1 | 11 |
| ECOWAS | 2 | NA | 2 | 101 | 2.0 | 11 |
| EEA | 4 | NA | 4 | 58 | 6.9 | 10 |
| EFTA | 1 | NA | 1 | 34 | 2.9 | 9 |
| EU | 7 | NA | 7 | 148 | 5.4 | 11 |
| GCC | 2 | NA | 2 | 54 | 3.7 | 8 |
| GUAM | 3 | NA | 3 | 57 | 5.3 | 11 |
| ICGLR | 3 | NA | 3 | 79 | 3.8 | 11 |
| IGAD | 1 | NA | 1 | 65 | 1.5 | 11 |
| IORA | 1 | NA | 1 | 24 | 4.2 | 10 |
| MGC | 4 | NA | 4 | 34 | 11.8 | 8 |
| MRC | 1 | NA | 1 | 17 | 5.9 | 9 |
| NAFTA | 4 | NA | 4 | 67 | 6.0 | 11 |
| NC | 2 | NA | 2 | 21 | 9.5 | 6 |
| OAS | 1 | NA | 1 | 58 | 1.7 | 9 |
| OECS | 2 | NA | 2 | 65 | 4.6 | 10 |
| OSCE | 4 | NA | 4 | 108 | 3.7 | 11 |
| PA | 6 | NA | 6 | 74 | 8.1 | 10 |
| PIF | 3 | 1 | 4 | 90 | 4.4 | 11 |
| SADC | 3 | NA | 3 | 54 | 5.6 | 11 |
| SCO | 3 | NA | 3 | 57 | 5.3 | 11 |
| SICA | 4 | NA | 4 | 125 | 3.2 | 11 |
| SPC | 1 | NA | 1 | 13 | 7.7 | 6 |
| UEMOA | 1 | NA | 1 | 52 | 1.9 | 10 |
| UNASUR | 1 | NA | 1 | 53 | 3.8 | 10 |

Abbreviations: AC, Arctic Council; ACC, Arab Cooperation Council; ACD, Asia Cooperation Dialogue; ACS, Association of Caribbean States; ACTO, Amazonian Cooperation Treaty Organization; AL, Arab League; ALADI, Latin American Integration Association; ALBA, Bolivarian Alliance for the Peoples of Our Americas; AMU, Arab Maghreb Union; ANDEAN, Andean Community; APEC, Asia-Pacific Economic Cooperation; ASEAN, Association of South East Asian Nations; AU, African Union; BEU, Benelux Economic Union; BIMSTEC, Bay of Bengal Initiaitve for Multi-Sectoral Technical and Economic Cooperation; BSEC, Black Sea Economic Cooperation; CACM, Central American Common Market; CAEU, Council of Arab Economic Unity; CALC, Latin American and Caribbean Summit on Integration and Development; CAREC, Central Asia Regional Economic Cooperation; CARICOM, Caribbean Community; CBSS, Council of the Baltic Sea States; CCTS, Cooperation Council of Turkic Speaking States; CE, Conseil de l'Entente; CEEAC, Communaute Economique des etats de l'Afrique Centrale; CEFTA, Cental European Free Trade Agreement; CELAC, Community of Latin American and Caribbean States ; CEMAC, Communaute Economique et monetaire de l'Afrique centrale; CENSAD, Community of Sahel-Saharan States; CEPGL, Economic Community of the Great Lakes Countries; CIS, Commonwealth of Independent States; CoE, Council of Europe; COMESA, Common Market for Eastern and Southern Africa; CSTO, Collective Security Treaty (Organization); EAC, East African Community; EAEU, Eurasian Economic Union; ECO, Economic Cooperation Organization; ECOWAS, Economic Community of West African States; EEA, European Economic Area; EFTA, European Free Trade Association; EU, European Union; G5S, G5 du Sahel; GCC, Gulf Cooperation Council; GGC, Gulf of Guinea Commission; GUAM, Organization for Democracy and Economic Development; ICGLR, International Conference on the Great Lakes Region; IGAD, Intergovernmental Authority on Develeopment; IOC, Indian Ocean Commission; IORA, Indian Ocean Rim Association; LCBC, Lake Chad Basin Commission; MERCOSUR, Mercado Commun del Sur; MGC, Mekong-Ganga Cooperation; MRC, Mekong River Commission; MRU, Mano River Union; MSG, Melanesian Spearhead Group; NAFTA, North American Free Trade Organization; NATO, North Atlantic Treaty Organization; NC, Nordic Council; OAS, Organization of American States; ODECA, Organization of Central American States; OECS, Organization of Eastern Caribbean States; OSCE, Organization for Security and Cooperation in Europe; PA, Pacific Alliance; PIF, Pacific Islands Forum; SAARC, South Asian Association for Regional Cooperation; SACU, Southern African Customs Union; SADC, Southern African Development Community; SCO, Shanghai Cooperation Organization; SEATO, Southeast Asia Treaty Organization; SELA, Latin American Economic System; SICA, Central American Integration System; SPC, Pacific Community; SPECA, UN Special Program for the Economies of Central Asia; UEMOA, West African Economic and Monetary Union; UNASUR, Union of South American Nations; WTO, Warsaw Treaty Organisation.

**eTable 3.** **The association between organizational characteristics, socioeconomic and demographic factors, health status and health system capacity and the growth in the health policy competency scope of regional organizations, Coefficient (95% CI with Bonferroni correction)**

|  | Regression with Two-way Fixed Effects | Regression with Time Fixed Effects | Regression with individual Fixed Effects |
| --- | --- | --- | --- |
| **Characteristics of regional organizations** |  |  |  |
| Macro region |  |  |  |
| Europe |  |  |  |
| Americas |  | 0.38 (-0.8, 1.56) |  |
| Asia |  | -1.11 (-2.15, -0.07) |  |
| Africa |  | -0.23 (-1.27, 0.8) |  |
| Age of regional organization |  | -0.04 (-0.08, 0.01) | -0.1 (-0.17, -0.03) |
| Founding year (Yes =1) | 5.06 (3.01, 7.12) | 2.68 (1.8, 3.57) | 4.92 (3.08, 6.76) |
| Number of member states | -0.08 (-0.49, 0.34) | -0.02 (-0.06, 0.02) | -0.05 (-0.34, 0.23) |
| Regional court (Yes=1) | -0.57 (-3.3, 2.17) | 0.19 (-0.95, 1.33) | 0.11 (-4.28, 4.49) |
| Consenus decision-making (Yes=1) | 0.57 (-2.17, 3.32) | -0.44 (-1.18, 0.29) | 2.35 (-1.01, 5.71) |
| Unanimous decision-making (Yes=1) | -1.44 (-5.34, 2.47) | 0.02 (-0.92, 0.97) | -2.23 (-6.68, 2.21) |
| Simple majority decision-making (Yes=1) | 0.43 (-2.81, 3.67) | -0.51 (-1.63, 0.61) | 1.36 (-2.63, 5.36) |
| Qualified majority decision-making (Yes=1) | 1.68 (-0.01, 3.37) | 0.17 (-0.83, 1.17) | 0.66 (-1.97, 3.29) |
| Policy scope in all fields | -0.01 (-0.07, 0.05) | 0.02 (0, 0.03) | -0.04 (-0.09, 0.01) |
| Policy fields | 1.78 (0.8, 2.76) | 0.34 (0.08, 0.59) | 1.9 (0.93, 2.87) |
| **Socioeconomic and demographic factors** |  |  |  |
| Average - Scoio-demographic Index | -5.33 (-12.84, 2.17) | 0.01 (-1.45, 1.48) | 0.32 (-5.31, 5.94) |
| CV - Scoio-demographic Index | -0.51 (-3.33, 2.31) | 0.41 (-0.34, 1.15) | -0.42 (-3.27, 2.43) |
| Average - GDP per capita (2020 USD) | 2.11 (-0.48, 4.7) | -0.14 (-1.02, 0.75) | 3.05 (0.24, 5.86) |
| CV - GDP per capita | 0.24 (-2.16, 2.63) | -0.03 (-0.58, 0.52) | 0.09 (-1.52, 1.69) |
| Average - KOF Globalization Index | -4.9 (-11.56, 1.76) | -0.3 (-1.64, 1.04) | -6.84 (-12.94, -0.74) |
| CV - KOF Globalization Index | -0.36 (-2.59, 1.86) | -0.4 (-1, 0.2) | -0.86 (-3.05, 1.33) |
| Average - Trade (million USD） | 0.55 (-0.5, 1.61) | -0.05 (-0.58, 0.49) | 0.08 (-0.63, 0.78) |
| CV - Trade | 1.71 (0.02, 3.39) | 0.18 (-0.55, 0.91) | 1.33 (-0.34, 3) |
| Average - Population of member states | -2.8 (-5.96, 0.36) | 0.19 (-0.09, 0.46) | -2.39 (-5.81, 1.03) |
| CV - Population of member states | -5.17 (-9.86, -0.49) | -0.46 (-1.24, 0.32) | -3.8 (-7.56, -0.04) |
| Average - Percentage of population aged 15-64 | 4.58 (-0.51, 9.68) | 0.89 (-0.34, 2.11) | 3.44 (-0.31, 7.19) |
| CV - Percentage of population aged 15-64 | -1.15 (-3.02, 0.72) | -0.12 (-0.52, 0.29) | -0.68 (-2.31, 0.96) |
| Average - Continuous | -0.36 (-1.86, 1.15) | 0.24 (-0.17, 0.65) | -0.09 (-1.51, 1.33) |
| CV - Continuous | -1.35 (-2.72, 0.02) | -0.08 (-0.79, 0.63) | -0.76 (-1.76, 0.23) |
| **Health status** |  |  |  |
| Average - Life expentancy at birth | -3.65 (-8.19, 0.9) | 0.35 (-1.01, 1.7) | -3.52 (-8.86, 1.81) |
| CV - Life expentancy at birth | 1.59 (-0.02, 3.2) | 0.05 (-0.51, 0.62) | 1.47 (-0.32, 3.25) |
| Average - Under-five mortality rate | 5.75 (0.07, 11.44) | -0.79 (-2.1, 0.52) | 5.85 (-1.02, 12.73) |
| CV - Under-five mortality rate | -2.9 (-5.8, -0.01) | -0.35 (-0.96, 0.27) | -2.71 (-5.24, -0.19) |
| Average - Age standardized moratality of all causes | -3.08 (-7.96, 1.81) | 0.72 (-0.54, 1.97) | -2.63 (-9.23, 3.98) |
| CV - Age standardized moratality of all causes | -1.09 (-3.13, 0.95) | 0.13 (-0.49, 0.74) | -1.33 (-2.99, 0.34) |
| **Health system capacity** |  |  |  |
| Average - Healthcare Access and Quality Index | 2.35 (-1.85, 6.55) | 0.2 (-0.89, 1.3) | -3.44 (-7.62, 0.74) |
| CV - Healthcare Access and Quality Index | 1.59 (0.02, 3.15) | -0.09 (-0.7, 0.52) | 1.19 (-0.67, 3.05) |
| Average - Hospital beds per 1000 people | -3.15 (-6.17, -0.13) | 0.18 (-0.52, 0.88) | -0.05 (-4.39, 4.29) |
| CV - Hospital beds per 1000 people | 0.67 (-2.15, 3.48) | 0.2 (-0.23, 0.64) | 0.16 (-1.73, 2.05) |
| Average - Health worker density | 1.16 (-1.27, 3.59) | -0.23 (-1, 0.54) | 1.04 (-5.05, 7.12) |
| CV - Health worker density | -2.17 (-4.1, -0.25) | 0.01 (-0.39, 0.41) | -1.05 (-2.84, 0.75) |

Poisson pseudo-likelihood regression with multiple levels of fixed effects were used for analysis. CV, coefficient of variation.
